# Practical lessons for phone-based assessments of learning

**DOI:** 10.1136/bmjgh-2020-003030

**Published:** 2020-07-22

**Authors:** Noam Angrist, Peter Bergman, David K Evans, Susannah Hares, Matthew C H Jukes, Thato Letsomo

**Affiliations:** 1 University of Oxford, Oxford, Oxfordshire, UK; 2 Young 1ove, Gaborone, Botswana; 3 Teachers College of Columbia University, New York, New York, USA; 4 Center for Global Development, Washington, DC, USA; 5 Center for Global Development, London, UK; 6 International Education Division, RTI International, London, UK

**Keywords:** health economics, public health, health services research

## Abstract

School closures affecting more than 1.5 billion children are designed to prevent the spread of current public health risks from the COVID-19 pandemic, but they simultaneously introduce new short-term and long-term health risks through lost education. Measuring these effects in real time is critical to inform effective public health responses, and remote phone-based approaches are one of the only viable options with extreme social distancing in place. However, both the health and education literature are sparse on guidance for phone-based assessments. In this article, we draw on our pilot testing of phone-based assessments in Botswana, along with the existing literature on oral testing of reading and mathematics, to propose a series of preliminary practical lessons to guide researchers and service providers as they try phone-based learning assessments. We provide preliminary evidence that phone-based assessments can accurately capture basic numeracy skills. We provide guidance to help teams (1) ensure that children are not put at risk, (2) test the reliability and validity of phone-based measures, (3) use simple instructions and practice items to ensure the assessment is focused on the target skill, not general language and test-taking skills, (4) adapt the items from oral assessments that will be most effective in phone-based assessments, (5) keep assessments brief while still gathering meaningful learning data, (6) use effective strategies to encourage respondents to pick up the phone, (7) build rapport with adult caregivers and youth respondents, (8) choose the most cost-effective medium and (9) account for potential bias in samples.

**Summary box**

- Assessing children and youth remotely is essential to mitigating the adverse short-term and long-term public health and education impacts of the COVID-19 pandemic, as well as future school closures due to health and other crises.
- There is existing literature on best practice strategies to carry out phone-based surveys of adults, on oral face-to-face testing of learning among children and youth, and on using technology to help community health workers identify ill or at-risk children. However, there is little evidence on assessing learning among children and youth over the phone.
- Pilot experience with phone-based testing among our team, together with experience with oral assessments and phone-based surveys, provides preliminary guidance to orient those who would assess learning for out-of-school children when face-to-face assessments pose a public health risk.

## Introduction

School closures around the world due to COVID-19—with more than 1.5 billion learners affected—pose the potential to add a second public health challenge to the pandemic.^1^ In the short run, school closures are associated with rises in adolescent pregnancy.^2^ School closures also lead to dropout, with adverse impacts on subsequent health behaviours and health status.^3^ To keep students engaged and learning, education systems have rolled out a wide variety of distance-learning platforms: television programmes, radio programmes, web-based instruction, phone tutorials from teachers and others.^4^ Existing studies have measured how much children are engaging with educational content.^5 6^ But how much are they actually learning? Students commonly fall behind during school closures^7 8^ and that can also increase dropout rates.^9^ Children do not lose learning equally: children from high-income families gain learning during school closures, whereas children from low socioeconomic backgrounds lose the equivalent of several months of learning.^10^

Ongoing research projects in low-income and middle-income countries, where internet access can be both limited and inconsistent, seek to evaluate student learning by phone during the COVID-19 school closures to avoid putting assessors and youth at risk. There is a limited history of phone-based behavioural and learning assessments. Several studies have assessed the validity of phone-based assessments of cognitive function among elderly patients,^11^ including one study of literacy assessments in adults.^12^ Other studies have enabled community health workers to assess and report child health.^13 14^ We are not aware of any published studies on direct learning or health assessments of children that were administered by phone. This article combines past research and experience with oral assessments, with ongoing piloting of phone-based assessments in a middle-income setting (Botswana) to propose a series of preliminary principles for the assessment of learning by phone.

Assessment by phone is a nascent field of research and much will be learnt in the current crisis and beyond.^15 16^ In addition to ongoing work in Botswana, authors of this paper are associated with efforts in Sierra Leone and Tanzania in partnership with the Center for Global Development and RTI International, and other teams in other countries are also implementing pilots. Not all learning can be assessed by phone; understanding which domains of learning can be assessed with validity and improving the quality of these assessments may open the door to a more cost-effective measurement of student learning even after schools reopen. This article seeks to integrate principles from the existing literature on face-to-face assessments with findings from a pilot study of phone-based assessments in Botswana to propose an initial set of guidance on which future research can build. These lessons also have applications for assessing child health by phone.

## Pilot phone-based and complementary assessments in Botswana

In our piloting effort, Young 1ove—one of the largest non-government organisations in Botswana—worked in partnership with the Ministry of Basic Education to collect over 10 000 phone numbers in schools in 4 out of 10 regions in Botswana before schools closed for lockdown. Since schools have been closed, caregivers and students have been contacted to participate in remote learning interventions rolled out as a randomised controlled trial in partnership with Columbia University and the Abdul Latif Jameel Poverty Action Lab.^17^

In this paper, we draw on two assessments from our work in Botswana, a phone-based assessment and a face-to-face assessment (from before schools shut down). The phone-based assessments were administered to 2250 students who were in grades 3–5 before schools shut down. They were conducted by over 70 former teacher aides (which we will subsequently refer to as ‘assessors’) who call households directly. Training for all assessors was conducted using voice notes and sharing of written material via WhatsApp. During the survey, assessors call a parent and request to speak to the child at the household. They request that the parent provide the child with privacy and make clear the questions are ‘low stakes’ in that they have no reward and consequences associated, in order to facilitate honest responses. Numeracy questions are then read out loud in order of difficulty of operation: addition, subtraction, multiplication and division. Word problems are texted and the child is asked to read them out loud. The questions are each timed with a maximum of 2 min to ensure uniformity across assessments. Finally, the child is asked to explain their work to ensure that they understand and provide another measure of independent response.

We complement our data from these phone assessments with data from face-to-face assessments, also collected in Botswana, but before schools were shut down for the pandemic. This included two assessments, both conducted in schools between February and March of 2020 with 1080 students in grades 3–5. In the first, classroom teachers evaluated a ‘problem of the day’. Specifically, one problem would be assigned to the whole class and students sit on the floor at arms-length from their classmates to ensure they respond individually. The teacher reads a problem out loud or puts it on the board. Students proceed to write the problem and their response in an individual booklet. When they are finished, they raise their hand and the teacher collects the booklet. After class, the teacher flips through all booklets and marks whether the problem was correct in a scoring sheet, as well as the type of problem using a defined scheme (eg, addition, subtraction; one-digit or two-digit; with or without borrowing and so on) as well as a subjective rate for the level of difficulty of the problem. The problems-of-the-day were administered daily for a period of 15 days, recorded in individual student booklets and compiled by student and class. To the same sample of students, we administered the more comprehensive Annual Status of Education Report (ASER) test of numeracy.^16^

**Figure 1:**
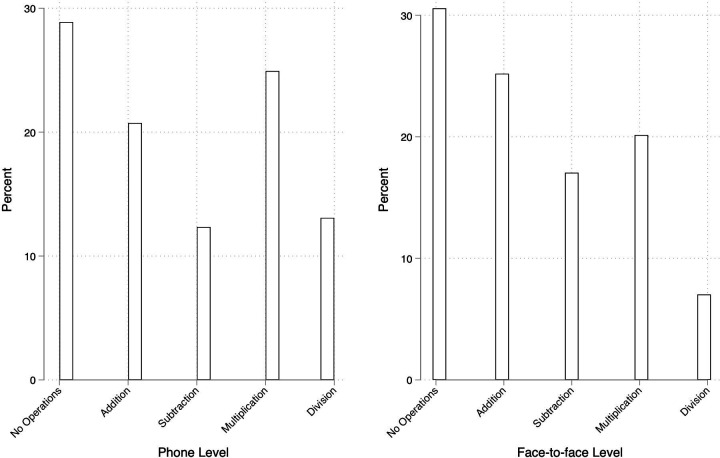
Percentage of students reaching each level of question difficulty (no operations, addition, subtraction, multiplication and division) in the phone-based sample and the face-to-face Annual Status of Education Report (ASER) test of the same content. These graphs are for the same regions and largely the same set of schools and grades, but they are not matched to the exact same cohort of students. They reveal a similar distribution of learning levels using the phone and face-to-face assessments at the population level in similar geographies and ages, and they increase our confidence in phone assessment. However, this is not yet a formal validity assessment. We plan to implement that in future phone-based assessments.

These data inform our preliminary practical lessons for future phone-based assessments. Comparing a sample of phone-based assessments with a more comprehensive ASER test demonstrates the promise of phone-based assessments for assessing basic skills. A sample of students from the phone-based assessment of numeracy reflects a similar skill level to the sample of students from the more comprehensive ASER assessment, administered face-to-face before schools shut down (figure 1).^18^ Moreover, we observe that responses cover the entire distribution of skills from not being able to do any operations to being able to do division, suggesting that even with simple operations, we can capture an array of student ability.

## Practical lessons for phone-based assessments

Although phone-based assessments are little studied, oral assessments of learning are commonly used directly with children and have much to teach about phone-based assessment. Orally administered tests are effective. Commonly used early grade assessments of reading and mathematics (such as ASER, Uwezo, and the Early Grade Reading and Math Assessments) are administered orally and have been validated.^19–22^ Instructions are presented orally and the response required from the participant is also oral. Some aspects of these assessments are, therefore, suitable for adaptation to phone surveys.

Conducting valid assessments by phone also presents challenges. We aimed to address some of these challenges in adapting oral assessments for administration by telephone, with suggestions drawn from experience and literature. We developed these lessons through an iterative process, in which team members shared their ongoing experiences with phone-based assessments and previous experience with and literature on oral assessments of learning to identify suggestions that we would recommend to any team beginning the process of phone-based assessments.

### Protect children

Much has been written about best practice in phone surveys,^23 24^ but few phone surveys gather data directly from children.^5^ It is vital to adapt face-to-face consent procedures and enumerator training to make sure that children and youth are not put in harm’s way in the process. For example, assessors can ensure that parents are aware that tests have no direct consequences for children (ie, these are low-stakes assessments), so that adults do not discipline children if they overhear low performance. Supervisors can also monitor a sample of calls to make sure assessors are interacting appropriately with children and youth. One way to accomplish this is to record a random sample of calls and have those automatically sent to supervisors. Furthermore, for assessments with young children, as the phones usually belong to parents who receive the call, there is almost always another person besides the child in the household who is aware of the assessment to provide a layer of accountability and oversight. Researchers should adapt general principles of research with children and youth for phone-based assessment.^25^

### Test the reliability and validity of your measures

Before rolling out an assessment, it is essential to ensure that it measures the specific skill that you want to measure—rather than, for example, general language skills—and does so reliably.^26 27^ Fortunately, this can be done at a fraction of the cost of the overall assessment. The simplest psychometric assessments examine the internal structure of the phone-based assessment, to examine the internal reliability (Cronbach’s Alpha),^28^ factor structure or item analysis, for example, using item response theory models. Such analyses help support the reliability of the tool and can identify problematic questions. Ideally, phone-based assessments should be validated against established face-to-face assessments. Such a test of concurrent validity is not possible while schools are closed and communities are locked down.

**Figure 2:**
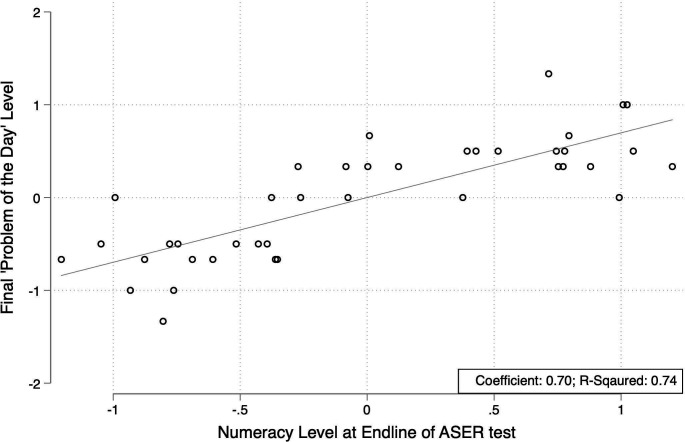
The relationship between student answers on “problem of the day” on the last day of class and average learning levels for the whole class after 15 days. Estimates were averaged at the class level within a school for a sample of 40 classes. Each individual student answered a ‘problem of the day’ in an individual booklet, which was compiled by the class teacher. If students answered problems correctly, then they progressed to more difficult items. At the end of 15 days, a more comprehensive multi-item oral assessment (the Annual State of Education Report, or ASER, assessment) was administered. In this figure, we compare the final problem-of-the-day level of difficulty with performance on the ASER test.

In Botswana, we approached current validity in a two-step process. The first step was to simplify an existing oral assessment in preparation for administration by phone. In this first stage, we assessed whether the simpler version of the face-to-face administered assessment was a valid proxy for the more comprehensive test. Specifically, we compared the ‘problem-of-the-day’ assessment, which is easily adapted to a phone-based assessment, with the more comprehensive ASER assessment.^19^ We correlated the difficulty level of the final problem-of-the-day with the comprehensive assessment taken shortly after and found a correlation of 69%. We further find a high R-squared value of 0.74 and an average relationship estimated by a multivariate regression of 0.70 when we control for school-level variation (figure 2). If replications demonstrated this relationship to be stable in the study population, then it would represent reasonable concurrent validity, a first step towards establishing overall construct validity for the test.^29^ The second stage of validation will be to test the concurrent validity of the phone-based assessments against face-to-face assessments.

### 3. Keep instructions simple and use practice items to ensure that respondents understand the exercise

As with other oral assessments, whatever you evaluate by phone bundles receptive language skills with the skill you are attempting to test. By phone, in the absence of visual cues, oral assessments are even more of a test of receptive language skills like vocabulary, listening and processing skills. Acknowledging and adjusting for this is particularly important in settings where different respondents speak different languages and may comprehend the language of instruction orally either better or worse than they read it. Simple instructions and practice items can ensure that more of the assessment is focused on the target skill. Data from our first wave of phone-based results reveal that over 75% of students understood and answered all the problems, and 24% understood all the problems but could only answer some. (Whether or not the student ‘understands’ the problem is a subjective judgement made by the assessor and so is less objective than the rate of correct answers.) The alignment of skill levels across the phone-based assessment and the face-to-face assessment (figure 1) suggests that with simple problems, like the arithmetic operations we are using, phone-based assessments can accurately capture student ability.

### 4. Some assessments will be more conducive to phone assessment than others

The elements of oral tests with minimal visual stimuli will be easiest to adapt to phone-based testing. For example, the ‘word problems’ subtest of the Early Grade Math Assessment involves only oral stimuli, whereas the ‘missing number’ subtest has a grid of numbers that may be hard to replicate on a phone display. That said, phone-based assessments can still incorporate text. Assessors could send a text message and ask the respondent to read the message aloud. In Botswana, we have tried sending simple texts for students in grades 3 through 5, such as ‘Katlego has 32 apples and organises them by PLACE VALUE. How many TENS does she have?’ We asked the child to read the problem out loud (assessing literacy skills) and then ask the child to solve the problem (assessing mathematics skills). We send the text message immediately before the phone call.

### 5. Keep it short

Home environments, particularly during lockdowns, may be crowded and noisy and phone calls can be frequently interrupted. Brief calls and assessments are more effective than longer calls. General guidance on conducting phone surveys suggests keeping them to 30 min.^23^ However, assessments with young people should be shorter. The Early Grade Reading Assessments (EGRA), a text-based but orally administered assessment, typically takes about 15 min per child when administered in full.^30^ Some evaluations have used a shorter version of EGRA, focusing on only three subtests.^31 32^ Calls for phone-based assessments in Botswana are taking between 15 and 20 min. About 50% of those calls are logistical (scheduling, organising the set-up of the house and building rapport) and the other 50% are dedicated to the assessment. Obviously, the best data on this would be derived from a series of tests of assessments of different lengths; in the absence of that, our experience may inform other teams in designing their assessments.

Although lengthy assessments may not be possible to conduct via phone, short assessments that are high frequency, simple and cheap can still be informative and easily conducted over the phone. With shorter assessments, if teams want estimates with the same level of statistical precision across the assessed students, then the sample size will need to rise.^33^ In face-to-face assessments in Botswana earlier this year—as described above—we observed a strong correlation between performance on the single ‘problem of the day’ and performance on the class assessment (figure 2). Although this was a face-to-face assessment, it demonstrates how simple tests like these, administered by phone, could indicate levels of learning loss or gain and therefore provide useful information for policy-makers and school systems attempting to mitigate the adverse health and educational effects of school closures.

### 6. Experiment with how to get people to pick up the phone

Piloting in Botswana revealed that the combination of a text message followed by a call yielded the highest pick-up rates. This is consistent with evidence elsewhere. Sending a text message to alert respondents to an upcoming call delivered the best responses in India.^34^ A programme in Liberia sent a text message 5 min before the call and found it helpful to boost answer rates.^13^ In Botswana, few people replied to texts alone and about 70% answered calls alone. Thus, a combination of the two may be most effective.

### 7. Establish rapport with adult phone owners and youth respondents

Respondents—both adults and youth—may be nervous, particularly during this time of global and local crisis. In many low-income and middle-income countries, conversations between adults and children are less common, as are interactions with strangers.^35^ Questioning oriented to children in some cultures is predominantly to obtain information that you are lacking, rather than to test the knowledge of another person. Rapport, explanation and examples can all help overcome these barriers. Having an advance call with an adult responsible for the target child can increase accuracy, honesty and a willingness to participate (beyond the obvious need for consent). In some cases, initial assessment instructions can be delivered through a caregiver with requests to put the child at ease. It is likely that phone-based tests will be challenging with children in early grades of primary school or in preschool.

### 8. Choose the most cost-effective approach

The full cost of phone calls to over 2250 households was about US$10 000, including airtime, personnel time, questionnaire design and piloting. This equates to about US$4.40 per child. To put this cost in context, the international assessment Progress in International Reading Literacy Study, which included Botswana in 2011, has standard fees for country participation. In Botswana, the costs are around US$250 000 and about 4000 students participated, yielding a cost of about US$62.5 per child. This is likely a lower bound, as country participation fees likely do not capture all costs. An average of school-based testing (for students in school) and home-based tracking (for students out of school), combined with classroom observations, as part of a randomised controlled trial in Liberia, cost US$150 per child.^36 37^

We use direct phone calls by assessors as access to phones is nearly universal and is a common denominator that is widely applicable across contexts. Another potentially lower-cost approach is the use of interactive voice response (IVR) calls, but they may have context-specific capability depending on the provider landscape. In Botswana, IVR infrastructure was not readily available. Lessons from direct calls reveal that about 50% of calling time is spent on logistics, including scheduling with parents and students, rescheduling, setting up at the household for the assessment and creating a conducive environment. This might imply that methods such as IVR, which might be cheaper and more scalable, might also have lower take-up rate as they might be harder to schedule reliably and may be less personal. Alternative methods and their relative cost-effectiveness are an empirical question for future work.

We hope this piece motivates further creative low-tech and cost-effective approaches to assessment. In the longer term, if consistently reliable methods and tools to measure learning by phone can be developed, they have the potential to disrupt the way we do measure learning, by enabling both high-frequency diagnostics and more cost-effective ways to assess learning outcomes.

### 9. Account for sample bias

A challenge to conducting phone-based assessments is that there may be systematic biases in sample selection. Although access to phones is nearly universal in Botswana, it is likely that households that do not respond to phone surveys differ from those that do. For example, non-responders may lack access to a phone or may live in a crowded household where it is difficult to speak quietly on the phone. The problem of sample bias applies both for validating phone-based assessments (via face-to-face assessments) and for data collection. Concurrent validity assessments may be flawed if a significant proportion of the face-to-face sample do not respond to phone-based assessments. The first approach to this problem is to document the bias. If socioeconomic indices are available for participating households, then these data can be used to understand how responders and non-responders differ. If bias is a concern, then a sample of non-responders can be selected for follow-up using different assessment methods (eg, asking a neighbour to lend them their phone) and the data from this subgroup could be weighted accordingly in final analyses.^38^

## Conclusions

Efforts to assess learning by phone are still new and so should not be used for high-stakes decisions around the future of individual students. However, understanding whether distance-learning efforts are leading to learning and identifying which groups of children are being most or least disadvantaged by being out of school should be a central part of the current response as well as any initiatives to help disadvantaged groups catch up once schools do reopen. That said, researchers should always remember that just as face-to-face assessments have limitations (high cost), so do phone-based assessments (more difficult to assess children with certain disabilities, like hearing loss). We have proposed an initial set of practical lessons for phone-based assessments based on the literature and the experience of piloting a phone-based assessment in Botswana. We are continuing to learn in our own practice: for example, we are experimenting with randomising assessors to avoid any systematic enumerator fixed effects and with randomising the set of questions posed to each child to measure both the reliability of constructs and to back out a measure of sampling error. Our hope is that future research will use, critique and validate these lessons and contribute to a communal effort to develop best practices in this area. Ensuring that children are learning, even when out of school, is crucial not only to their education but also to their health outcomes and the quality of their whole lives.
